# SARS-CoV-2 Vaccines, Vaccine Development Technologies, and Significant Efforts in Vaccine Development during the Pandemic: The Lessons Learned Might Help to Fight against the Next Pandemic

**DOI:** 10.3390/vaccines11030682

**Published:** 2023-03-17

**Authors:** Chiranjib Chakraborty, Manojit Bhattacharya, Kuldeep Dhama

**Affiliations:** 1 Department of Biotechnology, School of Life Science and Biotechnology, Adamas University, Kolkata 700126, West Bengal, India; 2 Department of Zoology, Fakir Mohan University, Vyasa Vihar, Balasore 756020, Odisha, India; 3 Division of Pathology, ICAR-Indian Veterinary Research Institute, Izatnagar, Bareilly 243122, Uttar Pradesh, India

**Keywords:** vaccines, COVID-19 pandemic, lesson learned, SARS-CoV-2

## Abstract

We are currently approaching three years since the beginning of the coronavirus disease 2019 (COVID-19) pandemic. SARS-CoV-2 has caused extensive disruptions in everyday life, public health, and the global economy. Thus far, the vaccine has worked better than expected against the virus. During the pandemic, we experienced several things, such as the virus and its pathogenesis, clinical manifestations, and treatments; emerging variants; different vaccines; and the vaccine development processes. This review describes how each vaccine has been developed and approved with the help of modern technology. We also discuss critical milestones during the vaccine development process. Several lessons were learned from different countries during the two years of vaccine research, development, clinical trials, and vaccination. The lessons learned during the vaccine development process will help to fight the next pandemic.

## 1. Introduction

The coronavirus disease 2019 (COVID-19) pandemic has been a shocking and miserable period, and it is now time to look back. COVID-19 originated in December 2019 when the first case was detected in Wuhan, China [1]. The WHO declared a Public Health Emergency of International Concern (PHEIC) on 30 January 2020, due to the rapid spread of the virus outside China. Subsequently, the WHO declared a pandemic on 11 March 2020 [2]. By June 2020, most large countries had been hit by the pandemic. The virus has infected more than 200 countries worldwide. A high case fatality rate (CFR) was found in the elderly male population. In this group, the average CFR was 1–7% [3]. Looking back at the country-wise CFR, the highest was reported in Mexico. The second-highest was recorded in Italy. Other significant CFRs were noted at the UK, Spain, France, and Russia [3]. There is a significantly higher risk of COVID-19 infection in patients with comorbidities, such as diabetes mellitus, cardiac problems, and hypertension [4]. As of 30 December 2022, more than 660 million cases of COVID-19 were identified, and more than 6.69 million deaths were reported. Several therapeutic and immunotherapeutic molecules have been identified to control the infection [5,6]. The therapeutic molecules include remdesivir, favipiravir, and dexamethasone [7,8]. The immunotherapeutic molecules include mavrilimumab and tocilizumab [7,9,10,11]. Numerous clinical trials have been performed to evaluate repurposed therapeutics against SARS-CoV-2.

Vaccinations play a significant role in global health. They help augment long healthy lives and life expectancy. Vaccination is a helpful method for preventing numerous deadly and infectious diseases. It has been noted to be one of the most significant ways to fight a pandemic [12,13]. Examples of its usefulness are the eradication of smallpox and polio [14,15]. Owing to the adoption of vaccination, the frequencies of numerous childhood diseases, such as measles and polio, have been considerably reduced [16,17]. Currently, influenza vaccination is widely administered every year to ensure safety against the seasonal flu [18,19]. Therefore, researchers have shown that vaccination is one of the most effective ways to control the spread of an infectious disease.

Several studies have been conducted on other kinds of coronaviruses, such as SARS-CoV and MERS-CoV [20]. Vaccines are yet to be developed and released for these. However, previous studies on SARS-CoV and MERS-CoV vaccine efforts have provided vital information regarding structural biology, molecular biology, and vaccine research. Of note are the spike glycoprotein’s antigenicity and the structures of these two viruses (SARS-CoV and MERS-CoV) [21,22]. The spike glycoprotein is a vaccine target for these two viruses. Scientists have also reported that the spike glycoprotein of SARS-CoV-2 is the most important target for vaccine development [23,24].

**Figure 1:**
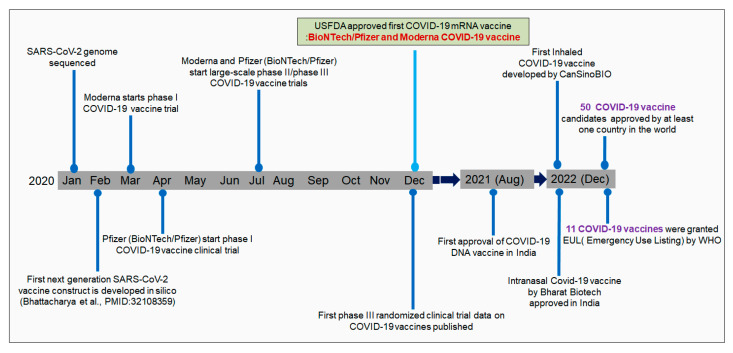
The timeline describes the different milestone achievements of vaccine development.

After identifying SARS-CoV-2 in China, Chinese researchers sequenced the virus’s genome. Zhang et al. sequenced the genome of SARS-CoV-2 at Fudan University. The genome sequence was immediately made publicly accessible in GenBank [25,26]. Genome sequencing initiated the immunoinformatic-based vaccine research to fight against SARS-CoV-2. Several researchers developed COVID-19 vaccine contracts using immunoinformatics [27]. Parallelly, pharmaceutical companies started vaccine development to fight the virus. First, Moderna initiated a clinical trial with mRNA-1273 from the Moderna vaccine in May 2020. Subsequently, Pfizer initiated a clinical trial with the vaccine candidates BNT162b1 and BNT162b2 with the collaboration of one German company, BioNTech [28]. Two mRNA vaccines (mRNA-1273 from Moderna and bnt162b2 from Pfizer) received initial approval (Emergency Use Authorization, EUA) by the USFDA and EMA at the end of 2020 or early 2021 (Figure 1) [29]. As of December 2022, 50 COVID-19 vaccine candidates have been approved by at least one country worldwide. At the same time, it has been reported that 201 countries have been vaccinating their populations with approved COVID-19 vaccines.

Similarly, till today, 11 COVID-19 vaccines have been granted an Emergency Use Listing (EUL) by the WHO [30]. Several vaccine candidates have been developed that have entered clinical trials over time [31]. In total, 242 vaccine candidates are in clinical development. Among them, 66 are in the phase-I developmental phase. Similarly, 72 vaccines are in phase-II, and 92 are in phase-III [30].

This review discusses SARS-CoV-2 vaccines, vaccine developmental technologies, and vaccine development efforts during the two years of the pandemic. We also discuss the key findings during vaccine development and vaccination. Several lessons were learned by different countries that might help to fight the next pandemic.

## 2. The First Approved Vaccines against SARS-CoV-2

The first approved vaccines were the Pfizer–BioNTech (vaccine: BNT162b) and Moderna (mRNA-1273) mRNA vaccines [29,32]. These two vaccines were approved by the EMA and FDA (USA) and have been granted EUA for use in the USA and Europe [29]. The first vaccine, Pfizer–BioNTech, received EUA from the USFDA on 11 December 2020 [33], and from the EMA on 21 December 2020 [34]. Simultaneously, the Moderna mRNA vaccine received EUA from the USFDA on 18 December 2020 [35]. Simultaneously, the vaccine (Moderna’s mRNA) received EUA by the EMA on 6 January 2020 [36] (Figure 1). Several vaccines have been approved in different parts of the world, such as CoronaVac, BBIBP-CorV, CoviVac, Covaxin, Oxford–AstraZeneca vaccine (ChAdOx1 nCoV-19), Sputnik V, the Johnson & Johnson vaccine, Convidicea, RBD-Dimer, and EpiVacCorona (Table 1). In an article published on September 2020, Parker et al. stated that approximately 200 vaccine candidates were involved in different developmental stages. Among these, some vaccine candidates entered phase-III clinical research [37].

## 3. The Vaccines Were Developed at Pandemic Speed

**Figure 2:**
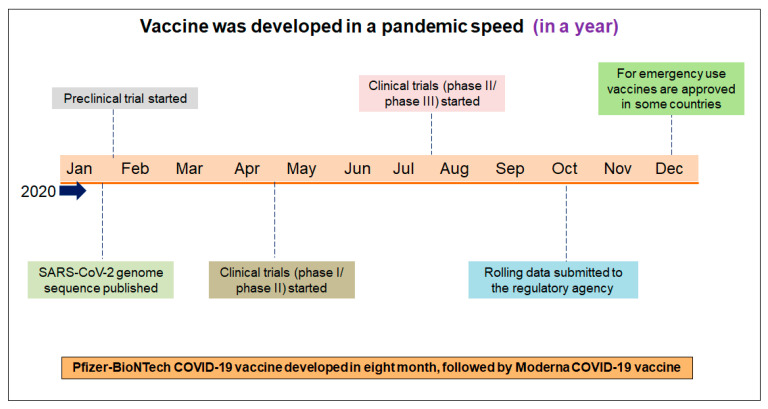
The different significant achievements of the first vaccine’s development and its approval process. The first vaccine (Pfizer–BioNTech mRNA) was developed within eight months. Several researchers call the speed of vaccine development “pandemic speed”.

Vaccines were rapidly conceptualized in the battle against COVID-19, and vaccine development was initiated against the virus. The vaccine candidates were developed first and then immediately entered into clinical trials from the experimental stage. The world has not seen such rapid vaccine development in recent years [50]. The vaccination development programs, followed by the first clinical trial, was concluded in December 2020. Therefore, the COVID-19 vaccine was developed faster than previously developed vaccines [51,52]. However, it should be noted that previous vaccine development experiences led to the faster development of COVID-19 vaccines. The Pfizer–BioNTech mRNA vaccine was developed and approved within eight months, and Moderna’s mRNA vaccine was developed and approved within a few days. These two vaccines were developed and received quick regulatory approval (EUA) during the pandemic (Figure 2).

However, previous research has helped to gain knowledge on SARS and MERS, and aided in the process of vaccine development against SARS-CoV-2. Researchers have been focusing on these two coronaviruses for years [50].

## 4. The COVID-19 Vaccine Platform

**Figure 3:**
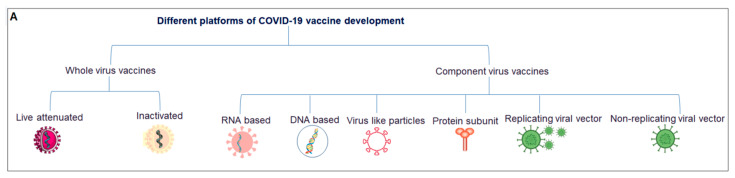

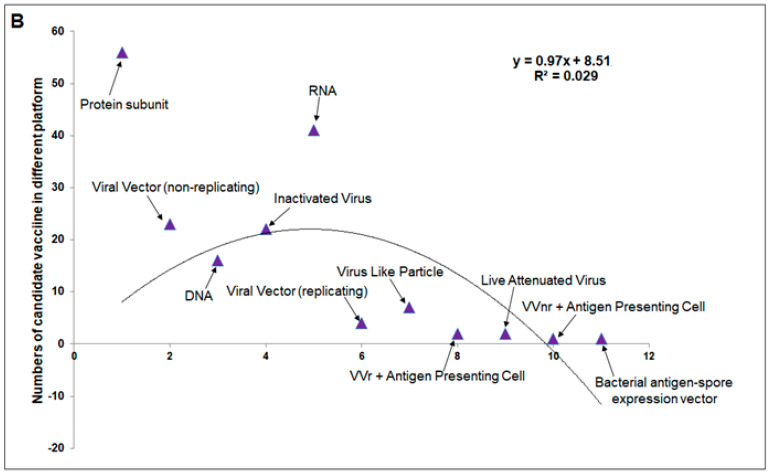

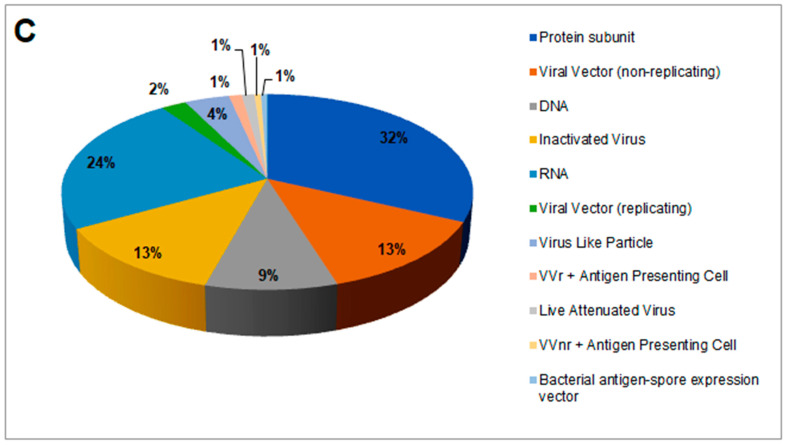
Different vaccine platforms and different clinical trials of vaccines. (**A**) A schematic chart that describes different vaccine platforms. (**B**) A statistical model was developed using the number of clinical trials. (**C**) Percentages of vaccine platforms described through a pie chart.

Considering all the vaccines developed in clinical trials, vaccines can be divided into two broad categories: whole-virus and component-virus vaccines. Whole-virus vaccines can be divided into two broad categories: live attenuated and inactivated. Similarly, component-virus vaccines can be divided into several broad categories: DNA-based, RNA-based, protein subunits, virus-like particles (VLPs)-replicated viral vectors, and nonreplicated viral vectors [53,54] (Figure 3A).

Currently approved vaccines are based on the inactivated virus (n = 11), DNA (n = 1), RNA (n = 4), protein subunits (n = 16), VLPs (n = 1), and nonreplicated viral vectors (n = 7) [55]; among these, 11 vaccines were EULs approved by the WHO.

A total of 175 vaccines are currently in different clinical phases of development, using protein subunits (n = 56), viral vectors (non-replicating; n = 23), DNA (n = 16), inactivated virus (n = 22), RNA (n = 41), viral vectors (replicating; n = 4), virus-like particles (n = 7), VVr + antigen-presenting cells (n = 2), live attenuated virus (n = 2), VVnr + antigen-presenting cells (n = 1), and bacterial antigen-spore expression vector (n = 1). We developed a statistical model using these vaccines with a second-order polynomial equation (Figure 3B) and determined the percentage of each (Figure 3C).

## 5. Different Approved Vaccines and Their Technological Platforms

The approved vaccines can be divided into four categories according to the type of vaccine platform utilized: mRNA vaccines, conventional inactivated vaccines, viral-vector vaccines, and protein-subunit vaccines (Table 1). Among these, two mRNA vaccines, four conventional inactivated vaccines, four viral-vector vaccines, and two protein-subunit vaccines have been approved. The authorized mRNA vaccines are the Moderna and Pfizer–BioNTech vaccines; conventional inactivated vaccines include CoronaVac, Covaxin, BBIBP-CorV, and CoviVac; viral-vector vaccines include Sputnik V, the Oxford–AstraZeneca vaccine, the Johnson & Johnson vaccine, and Convidicea; and protein-subunit vaccines include RBD-Dimer and EpiVacCorona. Moderna and Pfizer/BioNTech mRNA vaccines express the COVID-19 spike glycoprotein [56]. Vaccines from Oxford-AstraZeneca express spike proteins using adenovirus vector platforms [57]. Sinopharm developed a whole inactivated virus vaccine (BBIBP-CorV) using aluminum hydroxide as an adjuvant [58]. Similarly, a whole-virion inactivated virus vaccine was developed by BharatBiotech (Covaxin), and this vaccine was formulated with a TRL-7/TRL-8 agonist molecule that was adsorbed onto alum (AlgelorAlgel-IMDG) [47]. ZF2001 (RBD-Dimer) is a protein vaccine developed using the receptor binding domain (RBD) from the spike protein of the virus [56]. This vaccine uses aluminum as an adjuvant. EpiVacCoron is constituted with chemically synthesized epitopes conjugated to a recombinant protein carrier. This COVID-19 vaccine is adsorbed onto aluminum hydroxide [59]. Sputnik V is a viral-vector vaccine developed on a recombinant adenovirus platform using adenovirus 26 and adenovirus 5 (Ad26 and Ad5, respectively) vectors to express the spike protein of SARS-CoV-2 [41,60,61].

Inactivated whole vaccines are made through whole-virus vaccine preparations, such as CoronaVac (Sinovac), Covilo (Sinopharm), and Covaxin (Bharat Biotech). These vaccines have inactivated cells via chemical inactivation. Purification and mixing with particular compounds can be performed to stimulate immune cells. This specific compound is an adjuvant that amplifies immune responses. An example of an adjuvant is aluminum hydroxide [62]. It has been noted that heat-inactivated, irradiated, or chemically inactivated pathogens may lose their immunogenicity, and this platform is less efficient than live attenuated pathogen platforms [62].

Based on human or animal replication-defective adenovirus vectors, nonreplicated viral-vector vaccines, such as Covishield or Vaxzevria, have been approved for human use. Vaxzevria is by Oxford/AstraZeneca. On the other hand, Covishield is manufactured by two organizations: the Serum Institute of India and Fiocruz—Brazil. Covishield was developed and formulated by Oxford and AstraZeneca using a chimpanzee adenovirus encoding the SARS-CoV-2S glycoprotein [63,64]. Ad26.COV2.S is a replication-incompetent recombinant human adenovirus type 26 vector expressing the S protein, from Janssen/Johnson & Johnson, and has a very stabilized conformation [65].

## 6. Spike Protein Is the Center Point of Attraction in Vaccine Development

**Figure 4:**
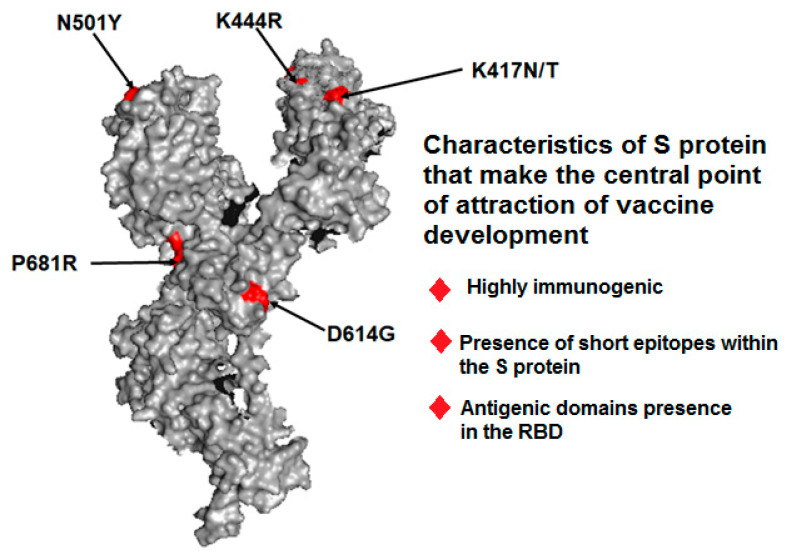
A spike protein’s 3D structure and its characters which make it the central point of attraction for vaccine development. Here, we depict some significant mutations in S proteins, such as P681R, N501Y, K444R, K41N/K, and D614G.

One key take-home message from pandemic vaccine development is that most vaccine development efforts were related to the use of the S protein, using which several preclinical studies were performed. The S protein is highly immunogenic. Most recent technologies, including immunoinformatics, have revealed its immunogenic nature. Structural proteins are the most common antigenic proteins. Martínez-Flores et al. reported the features of S glycoproteins, such as the presence of short epitopes within the spike and antigenic domains in the RBD [66]. Several other scientists have also reported that the SARS-CoV-2S protein is the most important target for vaccine development [23,24]. For the abovementioned reasons, the spike was chosen for vaccine development (Figure 4).

## 7. The Cost of the Vaccine

The cost of the vaccine is an essential factor for COVID-19 vaccination and is related to the worldwide accessibility of the vaccine. As of 2023, Moderna is selling its mRNA-1273 vaccine at a price of USD 25–37. BioNTech/Pfizer is selling its BNT162b vaccine at approximately USD 19 per dose. AstraZeneca is selling its vaccine at approximately USD 3–4. This company sells the vaccine to middle- and low-income countries on a non-profit basis to prioritize fighting the pandemic [67].

India is also producing low-cost vaccines [68]. The Serum Institute of India has agreed with Oxford University to produce more than one billion doses of the COVID-19 vaccine. They might supply inside the country and provide vaccines to low-and middle-income countries at a cost of USD 3 per dose [69]. Presently, the cost of the vaccine is USD 8–10 per dose.

## 8. Biggest Collaborative Effort of the 21st Century during Vaccine Development and Clinical Trials

Extensive collaborative efforts have been made during vaccine development and clinical trials. Several public–private partnerships have been formed [70]. Academic and government involvement was also noted at different levels to facilitate the assessment of endpoints and statistical analytical analysis. Industry participation with academia has also been noted. One example is the Oxford/AstraZeneca collaboration. Another collaboration is between AstraZeneca and the Serum Institute for manufacturing Covishield. We called during the early pandemic for a collaborative effort at different levels to fight the pandemic [71]. However, comprehensive teamwork and collaboration were observed during COVID-19 vaccine development. Another example of collaboration is the collaborative effort between three big organizations: Gavi, Coalition for Epidemic Preparedness Innovation (CEPI), and WHO. These three organizations aimed to deliver two billion vaccine doses globally by the end of 2021 [72]. They were likely to be successful in this direction.

## 9. The Real-World Data on COVID-19 Vaccine Effectiveness

Several studies have attempted to evaluate real-world vaccine effectiveness (VE) across the globe. COVID-19 phase-III trials have reported high VE for several vaccines against SARS-CoV-2. Pfizer-BioNTech’s mRNA vaccine’s VE was reported to be 95%; Moderna’s mRNA-1273 vaccine, 94.1%; Oxford-AstraZeneca’s ChAdOx1 nCoV-19 vaccine, 70.4%; and CoronaVac‘s absorbed inactivated vaccine, 50.7% [73,74] (Table 2). However, phase-III clinical trials have mainly enrolled young patients. Therefore, VE in elderly patients must be understood [75].

## 10. Reduced COVID-19 Vaccine Effectiveness against the Emerging Variants

Most of the leading COVID-19 vaccines, including Novavax, Johnson & Johnson, Pfizer/BioNTech, and Moderna, have shown reduced COVID-19 VE over time. Studies have shown that vaccine efficacy is reduced owing to the origin of emerging variants. Emerging variants can partially escape vaccines [89,90,91]. Several mutations were noted for immune escape and vaccine escape, and the vital mutations reported include D614G, P681R, E484K, N439K, K417N/T, K444R, and N501Y [89,92,93]. Furthermore, vaccines are less effective at protecting against infection from recently emerging viral variants, such as Omicron. Less effectiveness was noted even after the administration of a booster dose [81,94].

Some studies reported that VEs of the mRNA-based BioNTech, Pfizer vaccine, and mRNA-Moderna mRNA-1273 against alpha were similar to those against the previous variant [95,96]. However, most vaccines have reduced neutralization capacity against the Beta variant. The Sputnik V Ad26/Ad5, ChAdOx1 nCoV-19/AZD1222, CoronaVac, BNT162b2, mRNA-1273, and BBIBP-CorV vaccines showed reduced neutralization efficiency against Beta [97,98]. Similarly, the Omicron variant showed reduced neutralization capacity of immune sera elicited by vaccines, even after a booster [99] (Table 3).

## 11. Real-World Digital Platforms for Monitoring Every Country’s Status of COVID-19 Vaccination

After the rapid development of the COVID-19 vaccine, every country began vaccinating its population right away. They have developed strategies to vaccinate their populations. Most countries vaccinate their elderly populations first because they are the most vulnerable group in the country. US data show that they first vaccinated the elderly population [106]. However, several databases have been developed to determine the status of COVID-19 vaccination in every country. These databases provide information about each country’s vaccination status in terms of the “at least one dose” vaccinated population or fully vaccinated population, as a percentage or the number of vaccine-administered individuals. These databases also include data on the number of doses administered globally and the number of doses administered per day. Some critical databases are Our World in Data and COVID19-Vaccine Tracker. Most countries have their own databases to inform their vaccine status, such as the CDC in the USA and Co-WIN in India. The digital portal of India, Co-WIN, helped every Indian citizen receive the COVID-19 vaccine. The digital platform helped India conduct the world’s most significant vaccine drive [107]. However, the world has not seen this type of vaccine and vaccination effort before.

## 12. Approval of Intranasal Vaccine from Bharat Biotech and Inhaled Vaccine from CanSino Biologics: Will These Vaccines Be the Game Changers?

Recently, two next-generation COVID-19 vaccines have been approved by India and China: the Intranasal vaccine from Bharat Biotech and Inhaled Vaccine from CanSino Biologics Inc. (Tianjin, China), respectively [106,107,108,109,110]. These are mucosal vaccines, and both companies, have produced the vaccines through “viral vector” vaccines. CanSinoBIO used a recombinant viral vector platform (adenovirus from the Adenovirus Type 5 vector) to develop their vaccine. These vaccines are expected to induce mucosal immunity.

## 13. Effective Next-Generation Vaccine Design Research against Emerging Variants of SARS-CoV-2: A Recent Update

### 13.1. New or Modified Vaccine

The emerging SARS-CoV-2 variants, such as Delta and Omicron, have gained immune-evasion characteristics because of mutations in their genomes to overpower the existing COVID-19-vaccine-induced immune protection of neutralizing antibodies (nAbs), surpassing treatment with antibody-based therapies and resulting in breakthrough infections [20,93,111,112]. Meanwhile, it has been noted that these variants, Delta and Omicron, have higher transmissible properties than the wild strain. Certain questions arise. What happens if some variants with increased transmissibility gain higher virulence by acquiring sufficient mutations or recombination events? Will the strategy of producing vaccines using an ancestral method of concentrating on the viral spike sequence be continued? Moreover, will these ancestral vaccines protect against the upcoming variants with higher transmissibility or virulence? An advanced vaccine that can provide a broad range of protection against all emerging variants or upcoming variants of SARS-CoV-2 is needed. Simultaneously, we need to prepare for the next pandemic. Therefore, to keep pace with the continued emergence of SARS-CoV-2 variants, it is essential to update and modify currently available vaccines and design and develop new-generation vaccinations. New-generation vaccines include variant-specific vaccines [113], multivariant (multiple antigen-based) vaccines, mutation-proof vaccines, pan-coronavirus and universal vaccines [114], multi-epitope vaccines [115,116], CRISPR-based vaccines [117], artificial-intelligence-based vaccines [118,119], immunoinformatics- and immunomics-based vaccines [120], nanotechnology-based vaccines/nano-vaccines [19,121,122,123], nucleic acid-based and protein subunit-based vaccines, cytotoxic T cell based vaccines [124], and intranasal vaccines [125]. New-generation vaccines would be appropriately efficacious to tackle multiple emerging variants and future variants by preventing immune escape and rendering adequate protection against COVID-19 [109,123,124,125,126,127,128,129,130]. Hence, several scientists are attempting to develop modified or new vaccines that can provide broad protection against variants [129,131].

Considering the above, scientists are trying to develop a pan-coronavirus-protection vaccine as a futuristic approach. These scientists are developing strategies to protect against VOCs. In one clinical trial, researchers considered an mRNA vaccine based on the spike in the Wuhan strain or the spikes of rapidly upcoming VOCs (mRNA-1273/mRNA-1273.211/1273.351). These vaccines have been tested in booster cohorts and shown superior antibody titers against the variants. During the formulation of these vaccines, lipid nanoparticles have been used as vaccine delivery systems [132]. To develop second-generation vaccines to tackle multiple VOCs, an alphavirus-based replicating RNA vaccine expressing spike proteins of the original SARS-CoV-2 Alpha variant and recent VOCs has been designed. This vaccine uses a lipid-inorganic nanoparticle platform for in vivo delivery. This SARS-CoV-2 variant-specific replicating RNA vaccine protected against disease development in mice and Syrian Golden hamsters following challenge with heterologous VOC, eliciting strong neutralizing titers against homologous VOC. However, it demonstrated decreased titers against heterologous challenges and significantly reduced shedding of the infectious viruses. Such vaccine platforms could potentially be explored to target emerging VOCs [113].

On the other hand, researchers have developed adjuvanted RBD nanoparticles for pan-coronavirus protection. Saunders et al. (2021) formulated nanoparticles conjugated with the RBD of SARS-CoV-2. The vaccine was adjuvanted with alum and 3M-052 [133]. Several COVID-19 intranasal vaccines are being developed, which, apart from eliciting systematic immunity (both humoral and cell-mediated immunity), can also provide strong mucosal immunity via IgA antibodies. It can inhibit the virus at the mucosal level (the nasal cavity and lungs), prevent viral infection and replication, reduce virus shedding, and hinder disease development, thereby preventing further transmission and spread [11,134]. In this direction, researchers developed a vaccine for intranasal administration of virus-like particles (VLPs) exhibiting the RBD of SARS-CoV-2, which has been tested in a murine model. It can induce nAbs against the Wuhan strain of SARS-CoV-2 and other VOCs [135]. Recently, Wang et al. (2022) developed a multi-epitope peptide vaccine (UB-612) containing the S1-RBD-sFc protein and epitopes from spike (S2) proteins, membrane (M), and nucleocapsid (N) proteins. After phase-I or II clinical trials, this vaccine showed a robust booster outcome against VOCs, and a good safety profile. It also exhibited a broad range of T-cell and long-lasting B-cell immunity [116].

### 13.2. Nanoparticles Dotted “Mosaic” Vaccines with Different RBDs from SARS-CoV-2 and Coronaviruses

Recently, researchers at Caltech (California Institute of Technology) developed a nanoparticle-dotted vaccine that contains numerous RBDs from SARS-CoV-2. It can also contain RBDs from other coronaviruses. When a B cell recognizes multiple RBDs, it develops the capability to produce more antibodies. The vaccine can also trigger several memory B cells to fight future infections [136].

### 13.3. Emerging Vaccine against SARS-CoV-2 Using an Immunoinformtics Approach

Designing multi-epitope vaccines by employing immunoinformatic/computation-based approaches for SARS-CoV-2 seems promising, especially when exploring B and T cell epitopes. Immunoinformatic/computation-based multi-epitope vaccines could provide novel and putative vaccine constructs and potential candidates for developing vaccines to tackle COVID-19 [137]. Scientists have used antigenic epitopes from both the wild-type strain and mutated variants in this direction. We developed an in silico peptide-based vaccine construct using alternative antigenic epitopes from the Wuhan strain and other VOCs, which can boost immunity against these variants of SARS-CoV-2 [138]. A computational vaccine designed as a glycoprotein multi-epitope subunit vaccine candidate for old and new South African SARS-CoV-2 strains has been promising but requires further evaluation in animal models [115].

### 13.4. Other Recent Approaches

Scientists are also trying to develop mutation-proof COVID-19 vaccines. Wang et al. (2022) prepared a list of twenty-five mutations in the RBD. They developed nine commutation sets of mutations responsible for high infectivity, transmissibility, existing vaccine escape, and monoclonal antibody (mAb) escape [139].

### 13.5. Modern Tools and Technology for Next-Generation Vaccine Development against SARS-CoV-2 Variants

Similarly, researchers are applying modern tools and technologies, such as artificial intelligence (AI) and clustered regularly interspaced short palindromic repeats (CRISPR) technology, for next-generation vaccine design and development. Malone et al. (2020) applied AI to prepare a blueprint of antigenic epitopes to design universal COVID-19 vaccines. Using Monte Carlo analysis, they evaluated epitope hotspots for global epitope identification [140]. AI and machine learning techniques have facilitated the acquisition of sound knowledge on the genomic sequences of the SARS-CoV-2 virus and its variants (VOCs) and could aid in designing potential vaccines and drugs for tackling the COVID-19 pandemic [118,119]. Zhu et al. (2021) developed a universal platform for designing and developing SARS-CoV-2 vaccine candidates using multiplex bacteriophage T4 nanoparticles, which induced broad immunogenicity and rendered full protection against virus-challenge studies in a mouse model. In this study, CRISPR technology was applied to develop a robust nanoparticle platform [141]. Novel nano-vaccine construction using CRISPR technology might allow for quick exploitation of adjuvant-free, effective, nanoparticle-associated phage-based vaccines against any variants of SARS-CoV-2 or any future pathogen. Exploring CRISPR engineering of T4 bacteriophages to develop effective vaccines against SARS-CoV-2 and other emerging pathogens has been described in detail by Zhu et al. [141]. All these strategies are being used by researchers to provide protective immunity against SARS-CoV-2 and upcoming VOCs for next-generation vaccine development. Next-generation or modified vaccines will be safer and more effective than the current vaccines.

## 14. Limitations of COVID-19 Vaccines

Some vaccinated individuals developed severe forms of COVID-19. This occurred because of “vaccine escape” by SARS-CoV-2 variants. Due to mutations, several variants have developed in nature. Vaccine escape is a remarkable phenomenon in these variants. The latest SARS-CoV-2 Omicron variant and its subvariants are the most significant candidates for vaccine escape and contain several escape mutations [89,142,143,144,145,146,147,148]. Scientists are continuously trying to address this issue by creating next-generation vaccines with a broad range of immunity. These vaccines can produce a considerable number of antibodies and trigger several memory B cells to fight future infections. One example is the nanoparticle-dotted “mosaic” vaccine from Caltech [136]. Scientists are approaching this issue from different directions. We hope that the vaccine-escape problem will be addressed in the near future.

## 15. Take-Home Messages and Final Considerations

Here, we present several instances of vaccine development, which might be take-home messages and final considerations in this article. These instances serve as example guidelines to fight against future pandemics. First, after the emergence of SARS-CoV-2, a rapid and successful COVID-19 vaccine was developed within a year. This type of rapid vaccine development has never been reported. Vaccines for other diseases have been developed over several years. Therefore, this successful vaccine development strategy can be adopted to fight future pandemics. Second, collaborative efforts by public–private partnerships are crucial to the success of rapid vaccine development. Therefore, joint efforts are essential to fighting future pandemics. Third, vaccine research has laid a foundation for long-term effects. This research has been initiated in various directions, both basic and applied. Research on new vaccine technologies has also been conducted. At the same time, immunoinformatics-based research was initiated to map antigenic epitopes and to develop next-generation vaccine candidates, which will not only support responses to future pandemics, but also enrich vaccine research worldwide. Finally, scientists have noted that vaccine escape is a common phenomenon caused by both variants and subvariants. To protect against variants and subvariants, scientists have attempted to develop next-generation vaccines with broader and more durable protection mechanisms. Several researchers have initiated research on a “vaccine library” for different virus families to fight any future pandemic and to provide extensive preparation for future threats. However, ensuring equitable global access to vaccines is necessary, especially in middle- to low-income countries.

## 16. Conclusions

Finally, researchers are proud of the success of developing a COVID-19 vaccine. This is the first time a pandemic vaccine has gone from “bench to clinic” within a year. The impact of the vaccine development process will extend beyond the COVID-19 pandemic. The success of mRNA vaccines has encouraged the pharmaceutical community to invest in broader applications for various other infectious diseases. This technology can be applied to various metabolic diseases and cancers.

The time has come to make further efforts to collaborate at different levels. Research should develop “broad-spectrum” COVID-19 vaccines that can protect against VUMs, VOIs, and VOCs. At the same time, researchers must develop vaccines for all infectious viruses capable of triggering a pandemic. The lessons learned during COVID-19 vaccine development will help fight the future pandemics.

## Figures and Tables

**Table 1:** Different approved COVID-19 vaccines.

Sl. No.	Approved COVID-19 Vaccines	Developer of the Vaccines	Vaccine Type/Platform	Country of Origin	Storage Temperature (°C)	Dose	Reference
1.	Pfizer–BioNTech vaccine	BioNTech SE, Pfizer Inc.	Nucleoside-modified mRNA	Germany, USA	−70 ± 10	Two doses given three weeks apart	[38]
2.	Moderna vaccine (mRNA-1273)	United States National Institute of Allergy and Infectious Diseases, Moderna Inc., Biomedical Advanced Research and Development Authority, Coalition for Epidemic Preparedness Innovations	Nucleoside-modified mRNA	USA	−20 ± 5	Two doses given four weeks apart	[39]
3.	Oxford–AstraZeneca vaccine	AstraZeneca plc, University of Oxford, Coalition for Epidemic Preparedness Innovations	Modified adenovirus vector	Sweden, UK	2–8	Two doses given four to twelve weeks apart	[40]
4.	Sputnik V	Gamaleya Research Institute of Epidemiology and Microbiology	Modified adenovirus vector	Russia	≤−18	Two doses given three weeks apart	[41]
5.	Johnson & Johnson vaccine	Janssen Pharmaceuticals, Beth Israel Deaconess Medical Center	Modified adenovirus vector	Belgium, Netherlands	2–8	One dose	[42]
6.	Ad5-nCoV	CanSino Biologics, Beijing Institute of Biotechnology	Modified adenovirus vector	China	2–8	One dose	[43]
7.	BBIBP-CorV	Beijing Institute of Biological Products, Wuhan Institute of Biological Products, China National Pharmaceutical Group Corporation	Inactivated SARS-CoV-2	China	2–8	Two doses given three to four weeks apart	[44]
8.	ZF2001	Chinese Academy of Sciences, Anhui ZhifeiLongcom Biologic Pharmacy Co., Ltd.	Adjuvanted protein subunit	China	-	Three doses given in thirty days apart	[45]
9.	CoronaVac	Sinovac Biotech Ltd.	Inactivated SARS-CoV-2	China	2–8	Two doses given two weeks apart	[46]
10.	BBV152	Bharat Biotech International Limited, Indian Council of Medical Research	Inactivated SARS-CoV-2	India	2–8	Two doses given four weeks apart	[47]
11.	EpiVacCorona	State Research Centerof Virology and Biotechnology VECTOR	Peptide subunit	Russia	2–8	Two doses given three to four weeks apart	[48]
12.	CoviVac	Russian Academy of Sciences	Inactivated SARS-CoV-2	Russia	2–8	Two doses given two weeks apart	[49]

**Table 2:** Approved COVID-19 vaccines and their efficacy.

Sl. No.	Approved COVID-19 Vaccines	Efficacy	Reference
1.	Pfizer–BioNTech	Overall efficacy 95%	[76]
2.	Moderna vaccine (mRNA-1273)	Overall efficacy 94.5%	[39]
3.	Oxford–AstraZeneca	Overall efficacy 70% (64% after 1st dose)(70.4% after 2nd doses)	[77]
4.	Sputnik V	Overall efficacy 91.6%	[78,79]
5.	Johnson & Johnson	Overall efficacy 66.3% (72% in the USA)	[80]
6.	Ad5-nCoV	Overall efficacy 92% against severe COVID-19	[81]
7.	BBIBP-CorV	Severe COVID-19 showing efficacy 86–90%	[82]
8.	ZF2001	Overall efficacy of 90–96%	[83]
9.	CoronaVac	Overall efficacy 83·5% (after 2nd dose)	[84]
10.	BBV152	Overall efficacy 78%,severe COVID-19 disease: 93.4%	[85]
11.	EpiVacCorona	Overall efficacy 70–75%	[86]
12.	CoviVac	Overall efficacy 83% (after 2nd dose)	[87,88]

**Table 3:** Reduced vaccine efficacy of different significant COVID-19 vaccines against SARS-CoV-2 variants.

Sl. No.	COVID-19 Vaccine Name	Remarks	Reference
1.	Pfizer/BioNTech	In occurrence of Alpha variant the vaccine efficacy recorded to 81.5% from 95%.	[100]
		Vaccine efficacy reduced from 95 % to 6.7% encounter with Gamma variant	[101]
2.	Moderna vaccine (mRNA-1273)	The vaccine efficacy decreased to 60.9% from 94.5% in presence of Omicron variant	[102]
3.	Novavax (NVX-CoV2373)	The vaccine efficacy become lowering to 86% from 96% in existence of Alpha variant	[103]
		Vaccine efficacy reduced to 51% from 96 % to in occurrence of Beta variant	[104]
4.	Oxford–AstraZeneca	Vaccine efficacy was recorded 81%, and reduced 70% against Alpha variant	[100]
		Efficacy observed only 10% Beta variant	[105]
5.	Johnson & Johnson	The vaccine efficacy reduced in 52% for moderate infection, whereas for severe disease it shows 72% efficacy in USA, 64% efficacy in South Africa.	[103,104,105]

## Data Availability

Not applicable.
